# Random Copolymerization: An Efficient Strategy for Significantly Enhancing Photothermal Performance Through Synergistic Open-Shell Radical and TICT Effects

**DOI:** 10.3390/polym17040454

**Published:** 2025-02-09

**Authors:** Wenjin Xu, Haoran Tan, Yu Li, Xiaorui Ma, Haitao Xu, Dan Zhou, Qing Wan, Ruizhi Lv

**Affiliations:** 1 School of Material Science and Engineering, Nanchang Hangkong University, 696 Fenghe South Avenue, Nanchang 330063, China; xuwenjin1035@163.com (W.X.); 2301085600101@stu.nchu.edu.cn (H.T.); 202421021193@mail.scut.edu.cn (Y.L.); 2201085600056@stu.nchu.edu.cn (X.M.); 70244@nchu.edu.cn (H.X.); wanqingwork@163.com (Q.W.); 2 Key Laboratory of Jiangxi Province for Persistent Pollutants, Control and Resources Recycle, Nanchang Hangkong University, 696 Fenghe South Avenue, Nanchang 330063, China; zhoudan@nchu.edu.cn

**Keywords:** TICT, photothermal properties, random copolymerization strategy

## Abstract

Currently, photothermal (PT) polymers are gaining increasing attention in water evaporation, photocatalysis and photothermal therapy. However, high-performance PT polymers often require conjugated backbones and/or large fused units, which can impede non-radiative decay and lead to suboptimal PT performance. The development of general strategies for preparing high-performance PT polymers remains a significant challenge. In this paper, the high-performance donor–acceptor (D–A) random copolymers, named PBT4T-BBT-x (x = 0, 5, 10, 20 and 100), were fabricated by cross-mixing bithiophene donors with benzothiadiazole (BT) and benzodithiadiazole (BBT) acceptors. Notably, when the ratios of BT and BBT are finely tuned, the polymers exhibit significantly controllable open-shell radical effects and twisted intermolecular charge transfer (TICT) states. The synergistic effects of radicals and TICT states notably enhanced the PT performance of random copolymers. Specifically, when the proper ratios of BBT units are used, the photothermal conversion efficiency (PTCE) is remarkably increased from 21.7% to 58.5%, and the PT temperature obviously increases from 150 °C to 232 °C under 808 nm laser irradiation. Furthermore, the random copolymers exhibit good water evaporation rates. We propose that this strategy provides a valuable synthesis pathway for generating high-performance photothermal therapy and water evaporation materials.

## 1. Introduction

In recent decades, photothermal (PT) materials have garnered increasing attention due to their potential applications in seawater desalination, sewage treatment, phototherapy, anti-microbial applications and photocatalysis [1,2,3,4]. Organic photothermal molecules, in particular, have gained significant interest compared to inorganic photothermal materials due to their highly tunable molecular properties [5], including light absorption performance [6], molecular geometries [7], photothermal behaviors [8] and so on. In order to further improve their photothermal performance, the absorption abilities and nonradiative damping behaviors of the materials should be prioritized [9]. Notably, among the considerable efforts in enhancing light harvesting [10], D–A type molecules exhibit surprisingly broad and strong absorption due to the strong intramolecular charge transfer (ICT) effect and high planarity [11]. This has led to their widespread application in organic photovoltaics [12], fluorescence imaging [13] and high-performance photothermal materials [14]. Nevertheless, the enhancement of the ICT effect also results in significant radiation attenuation and noticeable fluorescence emission, which poses challenges in further improving the photothermal properties of these molecules [15,16].

To delve deeper into the photothermal properties of donor–acceptor (D-A) molecules, the attenuation pathway following light excitation must be considered [17]. On the other hand, although the suppression of non-radiative decay has been extensively explored [18,19], studies focusing on non-radiative attenuation remain scarce. Minimizing the bandgap and introducing triplet radicals represent important approaches to enhance non-radiative decay and improve photothermal performance [20]. By introducing a strong electron-withdrawing terminal group, Li et al. developed a planar acceptor–donor–acceptor (A–D–A) type molecule with a bandgap as narrow as 1.10 eV alongside open-shell radical properties. As a result, it demonstrated exceptional photothermal performance that exceeded 220 °C in the powder state [21]. Moreover, in recent years, a novel concept known as twisted intermolecular charge transfer (TICT) has emerged. Central to this concept is the fixed intramolecular charge transfer achieved through the distortion of the molecular skeleton between D and A blocks [22]. This rigidity inhibits energy transfer and significantly enhances non-radiative transition performance [23]. Huang Hui et al. reported donor–acceptor–donor (D–A–D) type photothermal materials based on terminal trianiline, which exhibited good absorption and photodynamic therapeutic properties in the infrared region [7]. Tang and his co-workers also reported the significant role of triphenylamine-based molecular rotors in enhancing nonradiative decay [24]. Furthermore, Lu et al. introduced dendritic triphenylamine groups to create D–A small molecules with strong TICT effects and exceptional photothermal properties [11]. However, the stability and synthesis complexity of free radical photothermal materials, as well as the relatively fixed structures of molecular-rotor-based materials, impede the further optimization of small-molecule photothermal materials [25].

Beyond small molecules, owing in part to their superior optical properties, thermal and chemical stability and impressive efficiency in film formation, the D–A conjugated polymers demonstrate distinctive benefits in medicine [26], energy [14] and related sectors [27]. The performance of these polymers can be readily optimized through adjustments in D and A composition [28], the implementation of side chain engineering [29], and the effects of substitution [30], enabling an easy fabrication of bespoke polymer materials. More importantly, using the random copolymerization strategies, the properties of random copolymers can be effectively and rapidly regulated, and the performance can also be conveniently optimized [31]. Nevertheless, owing to the unclear mechanism between the structure and non-radiative decay pathway of polymers, the further optimization of high-performance photothermal polymers remains challenging. Therefore, the exploitation of convenient and universal synthesis strategies of polymers with a suitable absorption range and strong non-radiative decay is of great importance for the development and application of high-performance photothermal polymer materials.

Herein, through the application of a random copolymerization strategy, a series of random copolymers named PBT4T-BBT-x (x = 0, 5, 10, 20 and 100) was successfully engineered by replacing certain quinoidal bithiophene (BT) units from the planar D–A copolymer PBT4T backbone with stronger electron-withdrawing units (bithienothiophene, BBT). The inclusion of BBT dramatically augments the light absorption potential in the infrared region, particularly in the secondary infrared (NIR II) zone, concurrent with a reduction in bandgap. The genesis of open-shell radicals, triggered by quinoidal groups, was validated using electron spin resonance (ESR) tests. Additionally, the random copolymerization-induced chain twisting provokes a shift in the polymer from the ICT to the TICT state. Fluorescence testing revealed that the polymer PBT4T-BBT-10, with 10% BBT content, exhibited significant fluorescence quenching, along with a red-shifted light emission, indicating the presence of pronounced TICT states. The synergistic effect of radicals and the TICT state significantly enhanced photothermal performance, which was further corroborated using photothermal performance tests. The PBT4T-BBT-5 powder reached temperatures over 230 °C when exposed to a 1.04 W cm^−2^ 808 nm laser. The photothermal conversion efficiency (PTCEs) was also remarkably improved from 21.7% to 58.5%. In addition, the viability of its photothermal performance and potential applications under conditions simulating sunlight were also validated, demonstrating promising water evaporation results.

## 2. Materials and Methods

### 2.1. Materials

All reagents and solvents, unless otherwise specified, were purchased from Energy Chemical (Anhui, China), Suna Tech (Suzhou, China), J&K Scientific (Beijing, China) and Derthon Optoelectronic Materials Co., Ltd. (Shenzhen, China). Toluene and chloroform were purchased from Jiangxi Xinphotoelectron Technology Co., Ltd. (Jiangxi, China)

### 2.2. Methods

#### 2.2.1. Instrument

UV-Vis absorption spectra were recorded on a Shimadzu UV-1900i spectrophotometer (Kyoto, Japan). Fluorescence spectra (PL) were measured with a Shimadzu RF-6000 spectrometer (Kyoto, Japan). Thermogravimetric analysis (TGA) was performed on a Diamond TG thermal balance under the protection of nitrogen at a heating rate of 10 °C/min. Differential scanning calorimetry (DSC) was recorded on a Seiko DSC-6220 differential scanning calorimeter (Chiba, Japan). Gel permeation chromatography (GPC) experiments were performed on an Agilent GPC 50 (Santa Clara, CA, USA) device. The ESR tests were performed on a Bruker EMXplus-6/1 instrument (Karlsruhe, Germany) with 9847 MHz microwave frequency.

#### 2.2.2. Cyclic Voltammetry

Cyclic voltammetry (CV) was performed on a CHI660C (version 8.03) potentiostat (Shanghai, China) equipped with electrochemical analysis system software and standard three-electrode configuration under an argon atmosphere at RT and a scan rate of 50 mV/s. A platinum rod, platinum wire and saturated calomel electrode were used as the working electrode, counter electrode and reference electrode in a 0.1 mol/L Bu_4_NPF_6_-acetonitrile solution, respectively. The ferrocene–ferrocenium (Fc/Fc+) redox couple is located 4.8 eV below the vacuum level. The *E*_HOMO_ and *E*_LUMO_ are calculated using Equations (1) and (2) (the redox potential of Fc/Fc+ is 0.4 V). The electrochemically determined band gaps were deduced from the difference between onset potentials from oxidation and the reduction of copolymers as noted in Equation (3).

(1) $$E_{HOMO}=-(E_{ox}+4.4)$$

(2) $$E_{LOMO}=-(E_{red}+4.4)$$

(3) $$E_{g}^{cv}=E_{ox}-E_{red}$$

#### 2.2.3. Photothermal Experiments

Here, 10 mg of polymer powder was pressed into a thin sheet with uniform thickness and exposed to an 808 nm laser (VCL808nmM1-4W, Beijing HongBlu-ray Technology Co., Ltd., Beijing, China). The polymer solution photothermal experiment involved exposing 1 mg/mL of dichlorobenzene (DCB) solution to 808 nm laser irradiation for 10 min. The temperature change was measured using an infrared thermal imager (Fotric 326L, Shanghai, China). The water evaporation experiments were performed using a xenon CEL-PF300L-3A (Beijing, China) light source. The xenon light source was calibrated with an optical power meter (CEL-NP2000-2(10)A, Beijing, China).

#### 2.2.4. Calculation of the Photothermal Conversion Efficiency

As previously reported [11], the photothermal conversion efficiency of polymers obtained its temperature–time cooling curve, which could be converted to a corresponding time–*lnθ* linear curve. Here, *θ*, which is defined as a dimensionless temperature, was introduced as follows:

(4) $$\theta =\frac{T-T_{surr}}{T_{max}-T_{surr}}$$

where *T* is the temperature of the DCB solution of polymers, *T_max_* is the maximum temperature of the cooling curve and *T_surr_* is the initial surrounding temperature.

As previously reported [11], the equation for calculating the photothermal conversion efficiency was derived as follows:

(5) $$\eta_{PT}=-\frac{\left(T_{max}-T_{surr}\right)\;ln\theta \;\Sigma_{i}m_{i}C_{p,i}}{tI(1-10^{-A_{808}})}$$

where $\frac{t}{ln\theta }\;$ is the slope of the fitted line on cooling curves in photothermal figures and *t* represents cooling time. In addition, *m_i_* is the mass and *C*_*p*,*I*_ is the heat capacity of the DCB solution, which is approximately the same as that of DCB. *I* is the laser power, and *A*_808_ is the absorbance of the polymer solution.

## 3. Results and Discussion

**Figure 1:**
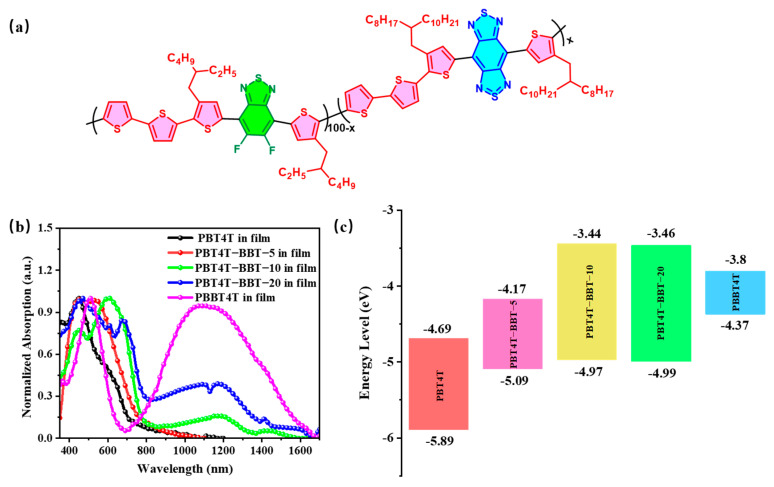
(**a**) The molecular structures, (**b**) UV-Vis absorption curves and (**c**) LUMO and HOMO levels of PBT4T, PBT4T-BBT-5, PBT4T-BBT-10, PBT4T-BBT-20 and PBBT4T.

**Scheme 1:**
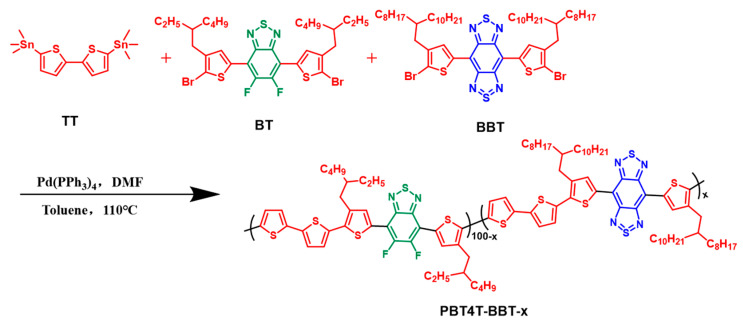
Synthesis routes of PBT4T-BBT-x (x = 0, 5, 10, 20, and 100).

The synthesis pathways of the target polymers are shown in Scheme 1; specific synthesis steps are shown in the Supporting Information. When 5,5-di(trimethyltin)dithiophene was mixed with 7-di(bromoethylhexylthiophenyl-2-)-difluoro-benzothiadiazole and/or 4,8-bis(5-bromo-4-(2-octyldodecyl)thiophenyl)-benzodithiadiazole in one pot, the random copolymers PBT4T-BBT-x could be easily synthesized through one-step Stille polymerization. Furthermore, by regulating the proportions of benzothiadiazole (BT) and benzodithiadiazole (BBT) units, the polymers PBT4T, PBT4T-BBT-5, PBT4T-BBT-10, PBT4T-BBT-20 and PBBT4T with different BBT contents can be obtained (Figure 1a). Furthermore, the Fourier infrared spectra of the polymers indicated the successful introduction of a BBT unit to the polymers (Figure S1). Moreover, according to the TGA and DSC measurements (Figures S2 and S3), these polymers exhibited strong stability, along with weak crystallinity.

### 3.1. UV-Vis-Absorption and Cyclic Voltammetry Measurements

In order to determine the optical properties of the random polymers, UV-Vis-*absorption* was assayed, and the results are presented in Figure 1b. Compared with the polymers in solution (Figure S4), both the maximum absorption peaks and the absorption edges of polymer films exhibited obvious red-shifted spectra. Moreover, the red-shifted spectra were all closely related to the BBT content. Compared with the copolymer PBT4T film, the maximum absorption peak of PBBT4T extended from 600 nm to 1080 nm, clearly entering into the NIR II zone. Moreover, the absorption edge of PBBT4T even approached 1700 nm, indicating potential PT applications in the NIR II region. In addition, when BBT was introduced, the absorption peaks of polymers at approximately 1100 nm were significantly increased, demonstrating that the random copolymers were well regulated. Notably, the introduction of a small amount of BBT (5%) had a minimal impact on its absorption curve. This phenomenon could be attributed to molecular skeleton distortion caused by random copolymerization. Furthermore, due to the PBT4T main unit, these polymers also exhibit broad absorption in the visible light region, indicating their effective utilization of solar energy. In addition, in the thin film state, all the BBT-based polymers exhibited a small absorption shoulder peak, which often suggests the generation of open-shell radicals [32,33]. Subsequently, cyclic voltammetry (CV) measurements were performed to assess the energy levels, and the results are summarized in Table 1 and Figure 1c. With the introduction of BBT, both lowest unoccupied molecular orbital (LUMO) and highest occupied molecular orbital (HOMO) energy levels significantly decreased, accompanied by a remarkable reduction in the bandgap; these findings are also consistent with the results obtained from the UV-Vis spectroscopy. Additionally, obvious double oxidation peaks were observed on the CV curves (Figure S5), indicating the potential existence of radicals. Furthermore, to gain insight into the radical properties, ESR measurements were also examined. The significant signals confirmed the presence of radicals in polymers (Figure S6), which could be attributed to the open-shell effects [34]. The results indicate that the significant open-shell pathway introduced by BT and BBT units likely contributes to radical generation, and the presence of the generated radicals indicate the potential high-performance photothermal properties of the copolymers [35].

### 3.2. Photoluminescence Measurements

**Figure 2:**
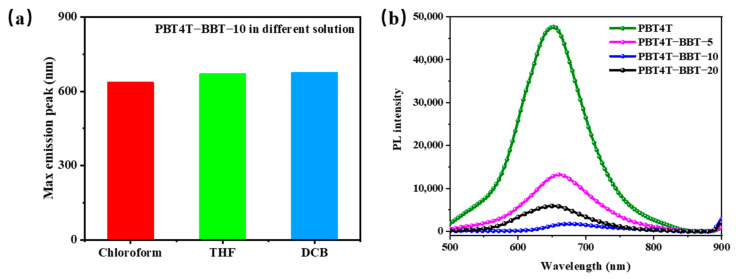
(**a**) The maximum emission peak of PBT4T-BBT-10 in different solvents. (**b**) Fluorescence spectra of PBT4T, PBT4T-BBT-5, PBT4T-BBT-10, PBT4T-BBT-20 and PBBT4T at a 450 nm excitation wavelength.

**Figure 3:**
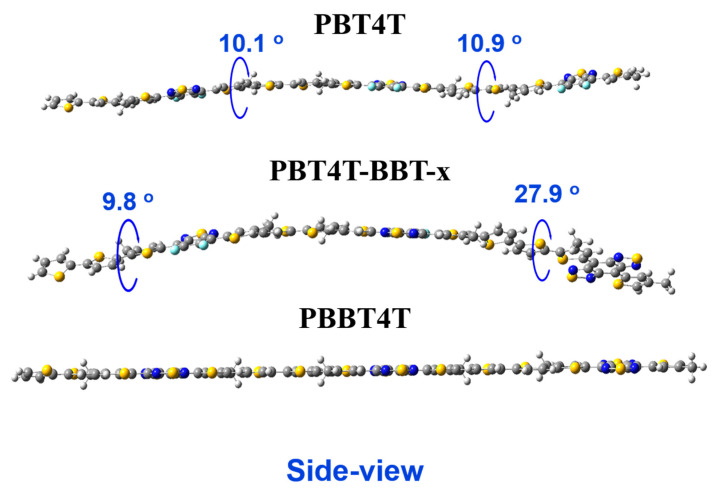
The calculated molecular structure of PBT4T, PBT4T-BBT-x and PBBT4T.

To obtain insight into the configuration of the random copolymers, photoluminescence (PL) measurements were performed. Frist, the solvent-dependent fluorescence of PBT4T-BBT-10 was obtained. Compared with chloroform, when tetrahydrofuran (THF) was used as the solvent, the maximum emission peak of PBT4T-BBT-10 was remarkably red-shifted from 637 nm to 671 nm. (Figure 2a) The peak was further red-shifted when PBT4T-BBT-10 was dissolved in DCB (the solvent with weaker polarity), which also demonstrated the existence of the TICT state of the random copolymers [36]. As shown in Figure 2b, when excited under 450 nm of light (the optimal excitation), PBT4T exhibited an obvious emission at 650 nm due to the strong D–A interaction between molecules. Furthermore, when BBT was introduced, a significant reduction in the emission curves was observed along with enhanced non-radiation relaxation, which can be attributed to the regulated TICT effect [19]. The results also indicate the destruction of the D–A planarity and the transition from the ICT state to the TICT state. The polymer structures were also calculated using three repeating units, and the results are shown in Figure 3. Quantum chemistry calculations using density functional theory (DFT) at the B3LYP/6-31G (d, p) level were employed to gain insight into the structure geometry and energy levels of PBT4T, PBT4T-BBT-x and PBBT4T. In order to simplify the calculations, methyl groups were used as substitutes for long alkyl chains; the results are shown in Figure 3 and Figure S7. Here, the PBBT4T copolymers exhibit excellent planarity with dihedral angles of 0.8 degrees. In contrast, the molecular backbone of the random copolymer undergoes significant distortion. These computational results once again provide evidence for the regulatory role of the random copolymer strategy in controlling the TICT state of polymers.

### 3.3. Photothermal Performance Measurements

**Figure 4:**
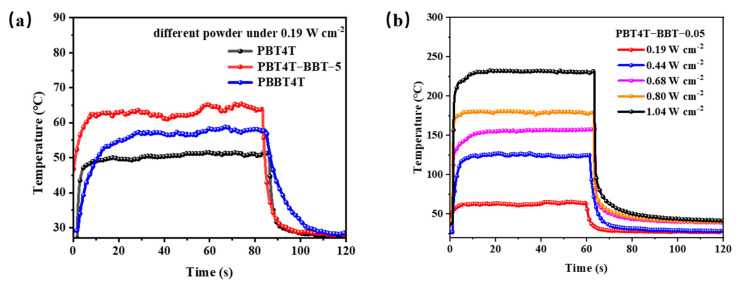
(**a**) Photothermal properties of PBT4T, PBT4T-BBT-5 and PBBT4T powder under an 808 nm laser (0.19 W cm^−2^). (**b**) Photothermal properties of PBT4T-BBT-5 powder under an 808 nm laser irradiation at different power irradiations.

The photothermal performances of the polymers were tested under different lasers. As shown in Figure 4a, the PT performance of PBT4T, PBT4T-BBT-5 and PBBT4T was examined under an 808 nm laser at 0.19 W cm^−2^. The polymer temperature of the polymers was 51.6 °C, 64.9 °C and 57.7 °C. These results indicate that the PT temperature was significantly improved by employing a random copolymerization strategy. Furthermore, the performance of random copolymers was then examined under 808 nm lasers of at 0.19–1.04 W cm^−2^. As depicted in Figure S8, it was evident that the PT temperature increased with higher power. Remarkably, the temperature of the PBT4T-BBT-5 polymer reached over 232 °C at 1.04 W cm^−2^ (Figure 4b), whereas the temperatures of PBT4T-BBT-10 and PBT4T-BBT-20 were slightly reduced (224 °C and 224 °C respectively). Probably due to the twist of the polymer main chain caused by the introduction of the third component, the photothermal performance was affected by both the enhancement of TICT and the reduction in radical effects. The competitive effect may have enabled PBT4T-BBT-5 to exhibit the best performance. Researches into the deep relationship between the random ratios and photothermal performance are also underway in our next works. Nevertheless, the BBT containing random copolymers still exhibited better PT-performance than PBT4T under an 808 nm laser at 0.19–1.04 W cm^−2^ lasers. This phenomenon indicates that the random copolymerization strategy affects photothermal conversion, potentially contributing to the disrupted planarity and the formation of the TICT state of the molecule due to the introduction of the third unit to the D–A copolymer backbone. In addition, the ESR examination also confirmed the generation of open-shell radicals. Therefore, the enhancement of photothermal performance can be attributed to the synergistic effect of the open-shell radicals and the TICT effect.

**Figure 5:**
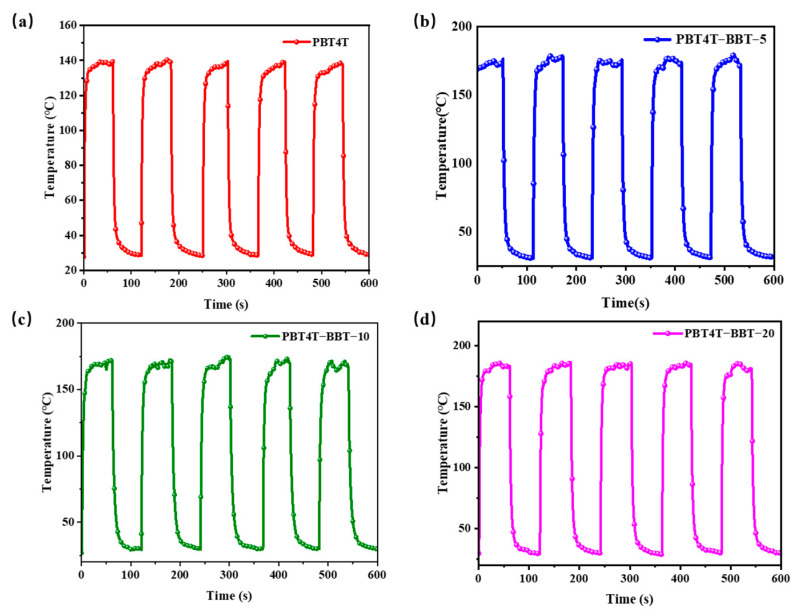
Photothermal properties of (**a**) PBT4T, (**b**) PBT4T-BBT-5, (**c**) PBT4T-BBT-10 and (**d**) PBT4T-BBT-20 powders irradiated with an 808 nm laser (0.80 W cm^−2^) for five heating/cooling cycles.

The PT stability of PBT4T, PBT4T-BBT-5, PBT4T-BBT-10 and PBT4T-BBT-20 powder was then examined. The results are shown in Figure 5. The temperatures of the four samples generally did not change during five cycles under 808 nm laser irradiation, indicating the excellent stability and repeatability of these materials.

**Figure 6:**
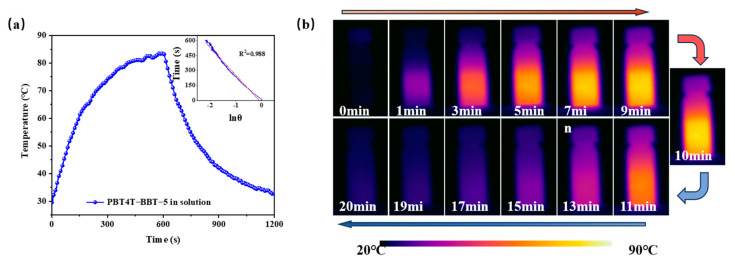
(**a**) Photothermal properties of PBT4T-BBT-5 in DCB solution under 808 nm laser (1 W cm^−2^) irradiation. The illustration shows the time–*lnθ* linear curve of the corresponding cooling process. The red line in (**a**) is the slope of the curve fit. (**b**) Infrared thermal imaging of PBT4T-BBT-5 in DCB solution under 808 nm laser (1 W cm^−2^) irradiation.

To learn more about their potential application abilities in PT, the PT performance of the polymers in solution were also assessed (Figure 6). Under 1 W cm^−2^ 808 nm laser irradiation, the temperature of PBT4T, PBT4T-BBT-5, PBT4T-BBT-10, PBT4T-BBT-20 and PBBT4T achieved values of 35.9 °C, 83.9 °C, 78.9 °C, 71.1 °C and 74.6 °C, respectively (Figure 6a and Figure S9), and the corresponding photothermal conversion efficiencies were 21.7%, 58.5%, 31.1%, 24.6% and 21.9%, respectively. These findings indicate that random strategy should remarkably enhance the PT performance. Other lasers with different wavelengths were also used to test the photothermal properties of the polymers. Figure S10 shows that when 5% to 20% ratios of BBT were introduced, the PT temperatures were significantly improved under 980 nm laser irradiation. However, due to the flexible nature of the polymer molecules, PBT4T-BBT-5, rather than PBT4T-BBT-10, displayed the highest maximum temperature. More importantly, due to the lower absorption around both 808 nm and 980 nm, PBT4T-BBT-5 demonstrated a remarkably high PTCE of 58.5% and 62.2%, respectively. These results all validate the excellent light conversion ability of the polymer in the NIR region, highlighting its great potential for applications in phototherapy and antibacterial application treatments.

To learn more about the PT processes of the random copolymers, additional PL measurements of the polymers in solution were obtained. Figure S11 shows that when 638 nm excitation light was used, all polymers exhibited upemission peaks around 380 nm. Although the emission light changed, the peaks still appeared. Moreover, compared with PBT4T, the introduction of the BBT unit resulted in a significant improvement in the emission signal on the emission curves. For example, when excited at 980 nm, PBT4T showed almost no peaks before 980 nm. However, the BBT-based polymer, PBBT4T, exhibited a small emission peak at 810 nm, demonstrating a slight emission induction effect of the BBT unit. More significantly, when a small number of BBT units were introduced, the peaks of the BBT-containing polymers, PBT4T-BBT-5 and PBT4T-BBT-10, rapidly increased. These results also suggest that the nonlinear optical process mainly occurred during the light harvest, leading to the sharply increased PT performance of the random copolymers.

### 3.4. Water Evaporation Measurements

**Figure 7:**
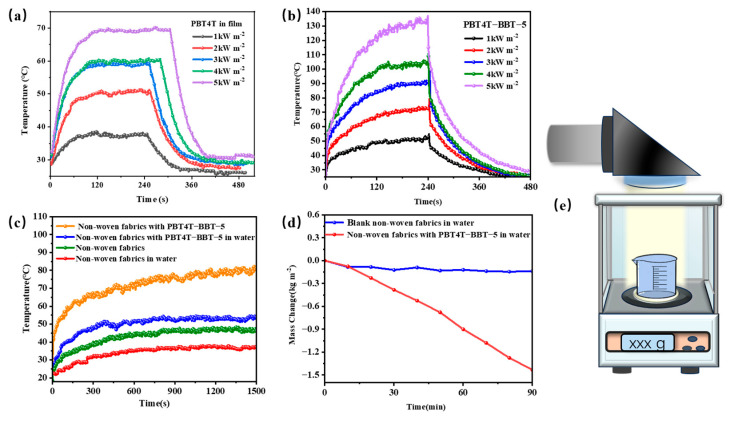
Photothermal properties of (**a**) PBT4T and (**b**) PBT4T-BBT-5 films under simulated sunlight at different light intensities (1~5 kW m^−2^). (**c**) The photothermal performance of blank non-woven fabric and non-woven fabric with PBT4T-BBT-5 covering 20 mL pure water under simulated sunlight irradiation (2 kW m^−2^). (**d**) Mass loss curves of 20 mL pure water covered with blank non-woven fabric and non-woven fabric with PBT4T-BBT-5 over 90 min under simulated sunlight irradiation (2 kW m^−2^). (**e**) Solar energy-driven water evaporation diagram.

In addition, the PT performances of the polymers under simulated sunlight were also studied to assess the solar energy transfer properties. As shown in Figure 7a,b, as the sunlight intensity increased from 1 kW m^−2^ to 5 kW m^−2^, the temperature of the PBT4T-BBT-5 film increased from 50 °C to 135 °C. On the contrary, the maximum temperature of PBT4T is approximately 70 °C. The PT should also be well regulated in random copolymers. Furthermore, we loaded PBT4T-BBT-5 onto a non-woven fabric and tested its photothermal performance under 2 kW m^−2^ sunlight. It is evident that the temperature of the non-woven fabric with PBT4T-BBT-5 rapidly increased to 80 °C whereas the blank non-woven fabric had a lower temperature of approximately 35 °C, close to room temperature (RT). When applied in a face solar evaporator, the non-woven fabric with PBT4T-BBT-5 maintained a temperature above 50 °C. Compared to blank non-woven fabric, the water evaporation rate of PBT4T-BBT-5-loaded non-woven fabric was significantly increased (η_5_ = 0.9599 kg m^−2^ h^−1^, η_blank_ = 0.1177 kg m^−2^ h^−1^) [37]. Additionally, the conversion efficiency of the PBT4T-BBT-5-loaded non-woven fabric was 33.1%, demonstrating the efficiency and convenience of the random copolymerization strategy in constructing efficient evaporation materials. (Figure 7c,d).

## 4. Conclusions

In this work, a series of random polymers with different numbers of BBT units were synthesized, and their PT performance was assessed. All of the random polymers, namely PBT4T, PBT4T-BBT-5, PBT4T-BBT-10, PBT4T-BBT-20 and PBBT4T, were easily prepared by mixing one donor (4T) and two acceptor (BT and/or BBT) monomers in a one-pot polymerization process. The introduction of BBT effectively regulates the absorption and energy levels of the polymers. More importantly, based on the PT measurements, the random strategy demonstrated extraordinary ability in enhancing PT performance. In the powder state, when 5% BBT was introduced, the stable temperature of PBT4T-BBT-5 was significantly increased to 232 °C under 808 nm laser irradiation at 1.04 W cm^−2^, whereas PBT4T only reached 152 °C. Moreover, PBT4T-BBT-10 and PBT4T-BBT-20 also displayed considerably higher PT temperatures than PBT4T, indicating that the random strategy should rapidly and effectively generate materials with superior PT properties. Subsequently, the performance of polymer solutions with lower mass fractions was also tested under lasers with different wavelengths. The random polymers maintained higher stable temperatures. Among them, PBT4T-BBT-5 exhibited the highest PTCE of 58.5%. According to the PL and ESR test results, the TICT effect and open-shell radicals were identified as key factors responsible for enhancing PT performance. Moreover, the PL measurements also revealed an up-emission effect under laser irradiation, contributing to the remarkable increase in PTCE of PBT4T-BBT-5 under NIR lasers. Furthermore, the polymers were also applied in water evaporators, demonstrating an excellent water evaporation efficiency of 0.9599 kg m^−2^ h^−1^. This work provides a convenient and rapid strategy to develop phototherapy and water evaporation materials with excellent and well-tuned near-infrared or solar photothermal properties. It also suggests the broad potential for applications in biomedical and photothermal fields.

## Figures and Tables

**Table 1:** The absorption and energy levels of PBT4T, PBT4T-BBT-5, PBT4T-BBT-10, PBT4T-BBT-20 and PBBT4T.

Polymers	λ_max_ (nm)	HOMO_CV_ (eV)	LUMO_CV_ (eV)	*E*_g_ (eV)
PBT4T	440	−5.89	−4.69	1.2
PBT4T-BBT-5	455	−5.09	−4.17	0.92
PBT4T-BBT-10	600	−4.97	−3.44	1.53
PBT4T-BBT-20	471	−4.99	−3.46	1.53
PBB4T	1080	−4.37	−3.80	0.58

## Data Availability

Data are contained within the article or Supplementary Materials. The original contributions presented in this study are included in the article or Supplementary Materials. Further inquiries can be directed to the corresponding author.

## Supplementary Materials

The following supporting information can be downloaded at: https://www.mdpi.com/article/10.3390/polym17040454/s1, Figure S1: Fourier infrared spectra of PBT4T, PBT4T-BBT-5, PBT4T-BBT-10, PBT4T-BBT-20 and PBBT4T; Figure S2: The TGA curves of PBT4T, PBT4T-BBT-5, PBT4T-BBT-10, PBT4T-BBT-20 and PBBT4T; Figure S3: DSC curves of PBT4T, PBT4T-BBT-5, PBT4T-BBT-10, PBT4T-BBT-20 and PBBT4T; Figure S4: The UV-vis absorption curves of PBT4T, PBT4T-BBT-5, PBT4T-BBT-10, PBT4T-BBT-20 and PBBT4T in film and solution; Figure S5: The CV curves of PBT4T, PBT4T-BBT-5, PBT4T-BBT-10, PBT4T-BBT-20 and PBBT4T; Figure S6: ESR spectrum of (a) PBT4T, (b) PBT4T-BBT-5 and (c) PBBT4T; Figure S7: HOMO and LUMO energy level diagrams of (a) PBT4T, (b) PBT4T-BBT-x and (c) PBBT4T; Figure S8: (a) PBT4T, (b) PBT4T-BBT-10, (c) PBT4T-BBT-20 powder under 808nm laser irradiation at different power irradiation, (d) Infrared thermal imaging under 808 nm laser at 0.68 W cm-2 of PBT4T-BBT-5 powder; Figure S9: Photothermal properties of (a) PBT4T, (b) PBT4T-BBT-5, (c) PBT4T-BBT-10, (d) PBT4T-BBT-20 and (e) PBBT4T under 808nm laser irradiation (1 W cm-2). The illustration shows the time-ln θ linear curve of the corresponding cooling process; Figure S10: Photothermal properties of (a) PBT4T, (b) PBT4T-BBT-5, (c) PBT4T-BBT-10 (d) PBT4T-BBT-20 and (e) PBBT4T under 980 nm laser irradiation (1 W cm-2). The illustration shows the time-ln θ linear curve of the corresponding cooling process; Figure S11: Fluorescence spectra at (a) 638 nm and (b) 980 nm excitation wavelength of PBT4T, PBT4T-BBT-5, PBT4T-BBT-10, PBT4T-BBT-20 and PBBT4T; Figure S12: GPC trace of PBBT4T; Table S1: GPC trace analysis of PBBT4T in Figure S12; Table S2: GPC trace analysis of PBT4T; Table S3: GPC trace analysis of PBT4T-BBT-10.
