# Supplementary material for: Random Copolymerization: An Efficient Strategy for Significantly Enhancing Photothermal Performance Through Synergistic Open-Shell Radical and TICT Effects
Source: Polymers (Basel). 2025 Feb 9;17(4):454. doi: 10.3390/polym17040454 (PMC11859739; doi:10.3390/polym17040454)
Supplement: Supplementary file 1 [file polymers-17-00454-s001.zip › polymers-3435856-supplementary.pdf]

Article

Random Copolymerization: An Efficient Strategy for Significantly Enhancing Photothermal Performance through Open-Shell Radical and TICT Synergistic Effects

Wenjin Xu [1,†], Haoran Tan [1,†], Yu Li [1], Xiaorui Ma [1], Haitao Xu [1], Dan Zhou [2], Qing Wan [1] and Ruizhi Lv [1,*]

[1] School of Material Science and Engineering, Nanchang Hangkong University, 696 Fenghe South Avenue, Nanchang 330063, China; xuwenjin1035@163.com (W.X.); 2301085600101@stu.nchu.edu.cn (H.T.); 202421021193@mail.scut.edu.cn (Y.L.); 2201085600056@stu.nchu.edu.cn (X.M.); 70244@nchu.edu.cn (H.X.); wanqingwork@163.com (Q.W.)

[2] Key Laboratory of Jiangxi Province for Persistent Pollutants, Control and Resources Recycle, Nanchang Hangkong University, 696 Fenghe South Avenue, Nanchang 330063, China; zhoudan@nchu.edu.cn

* Correspondence: lvruizhi@nchu.edu.cn

† These authors contributed equally to this work.

Experimental Section

Synthesis of PBT4T

Monomers of 5,6-difluoro-4,7-bis-(5-bromo-4-(2-ethylhexyl)-2-thienyl)-2,1,3-benzothiadiazole (BT) (144.4 mg, 0.2 mmol) and 5,5-di(trimethyltin)dithiophene (98.4 mg, 0.2 mmol) were added to a double-mouth round-bottomed flask, followed by 30 mL toluene, catalyst $Pd(PPh_3)_4$ (11.6 mg, 0.01 mmol) and 10 mL N, N-dimethylformamide (DMF). After sealing, the reaction unit is vacuumed through nitrogen for three times to completely replace the air. The reaction system is then heated to 110 °C. 20 minutes later, the color of the reaction system turns dark blue, this usually signals the end of the reaction. Then a small amount of solution is injected into methanol with a syringe to monitor the reaction. If precipitated particles are observed, the reaction ends. The crude reaction product was precipitated in methanol, and the filtered initial product was purified with ethanol, petroleum ether and ethyl acetate in a Soxhlet extractor. The products were subsequently transferred to a single-mouth flask with chloroform. The solvent was then removed via rotary evaporation, and the resultant polymer was subjected to drying for 48 h in a vacuum drying oven at 60 °C. Yielding PBT4T as a black solid (69.2 mg, 28.5%).

Synthesis of PBT4T-BBT-5

It was prepared by the same procedure as described above, starting with BT (136.5 mg; 0.19 mmol), monomer 4,8-bis(5-bromo-4-(2-octyldodecyl)thiophen-2-yl)-benzo[1,2-c;4,5-c']bis[1,2,5]thiadiazole (BBT) (10.8 mg; 0.01 mmol), and monomer 5,5-di(trimethyltin)dithiophene (98.4 mg; 0.2 mmol), followed by 30 mL toluene, catalyst $Pd(PPh_3)_4$ (11.6 mg; 0.01 mmol) and 10 mL of N; N-dimethylformamide (DMF). Yielding PBT4T-BBT-5 as a black solid (62.7 mg, 25.5%).

Synthesis of PBT4T-BBT-10

It was prepared by the same procedure as described above, starting with BT (129.4 mg; 0.18 mmol), BBT (21.5 mg; 0.02 mmol), and monomer 5,5-di(trimethyltin)dithiophene (98.4 mg; 0.2 mmol), followed by 30 mL toluene, catalyst $Pd(PPh_3)_4$ (11.6 mg; 0.01 mmol) and 10 mL of N; N-dimethylformamide (DMF). Yielding PBT4T-BBT-10 as a black solid (100.0 mg, 40.1%).

Synthesis of PBT4T-BBT-20

It was prepared by the same procedure as described above, starting with BT (115 mg; 0.16 mmol), BBT (43.1 mg; 0.01 mmol), and monomer 5,5-di(trimethyltin)dithiophene (98.4 mg; 0.2 mmol), followed by 30 mL toluene, catalyst $Pd(PPh_3)_4$ (11.6 mg; 0.01 mmol) and 10 mL of N, N-dimethylformamide (DMF). Yielding PBT4T-BBT-20 as a black solid (92.9 mg, 36.2%).

Synthesis of PBBT4T

It was prepared by the same procedure as described above, starting with BBT (215.5 mg; 0.2 mmol), and monomer 5,5-di(trimethyltin)dithiophene (98.4 mg; 0.2 mmol), followed by 30 mL toluene, catalyst $Pd(PPh_3)_4$ (11.6 mg; 0.01 mmol) and 10 mL of N, N-dimethylformamide (DMF). Yielding PBBT4T as a black solid (163.2 mg, 52%).

Calculation of the efficiency for solar to vapor generation

The conversion efficiency η of solar energy in photothermal assisted water evaporation was calculated as the following formula.[1]

$$\eta = \frac{\dot{m}h_{LV}}{C_{opt}P_0} \tag{6}$$

Where $\dot{m}$ refers to the mass flux (evaporation rate) of water, h_{LV} refers to the total liquid-vapor phase-change enthalpy (i.e., the sensible heat and the enthalpy of vaporization (i.e., $h_{LV} = Q + \Delta h_{vap}$)), Q is the energy provided to heat the system from the initial temperature to a final temperature T, Δh_{vap} is the latent heat of vaporization of water. P_0 is the nominal solar irradiation value of 1 kW m^{-2} and $Copt$ represents the optical concentration. The calculations of heat and enthalpy were as follows:

$$Q = C_{liquid} \times (T - T_0) \tag{7}$$
$$\Delta H_{vap} = Q_1 + \Delta H_{100} + Q_2 \tag{8}$$
$$Q_1 = C_{liguid} \times (100 - T) \tag{9}$$
$$Q_2 = C_{vapor} \times (T - 100) \tag{10}$$

In this paper, the specific heat capacity of liquid water (C_{liquid}) is a constant of 4.18 J (g °C)$^{-1}$; the specific heat capacity of water vaper (C_{vapor}) is a constant of 1.865 J (g °C)$^{-1}$; ΔH_{100} is the latent heat of vaporization of water at 100 °C, taken to be 2260 kJ kg^{-1}. The surface temperature of the PBT4T-BBT-5 loaded non-woven fabrics was 54.4 °C during the evaporation process, therefore T is 55 °C. As the above formulas:

$$Q = C_{liquid} \times (T - T_0) = 4.18 \times (54.4 - 25.9) = 119.13\ kJ\ kg^{-1}$$
$$\Delta H_{vap} = Q_1 + \Delta H_{100} + Q_2$$
$$= 4.18 \times (100 - 54.4) + 2260 + 1..865 \times (54.4 - 100)$$
$$= 2365.564\ kJ\ kg^{-1}$$
$$H_{LV} = Q + \Delta H_{vap} = 119.13 + 2365.564 = 2484.694\ kJ\ kg^{-1}$$
$$\dot{m} = 0.9599\ kg\ m^{-2}\ h^{-2}$$
$$P_0 = 2000\ W\ m^{-2}$$
$$C_{opt} = 1$$
$$\eta = \frac{\dot{m}h_{LV}}{C_{opt}P_0} = 33.1\%$$

As a result, the evaporation efficiency of 33.1% was determined for PBT4T-BBT-5.

Detailes of quantum chemistry calculation

The molecular geometries were optimized by Gaussian09 on the calculation level of B3LYP/6-31G (d, p) under normal convergence criteria with consideration of none solvent environment.

Additional Table and Figures

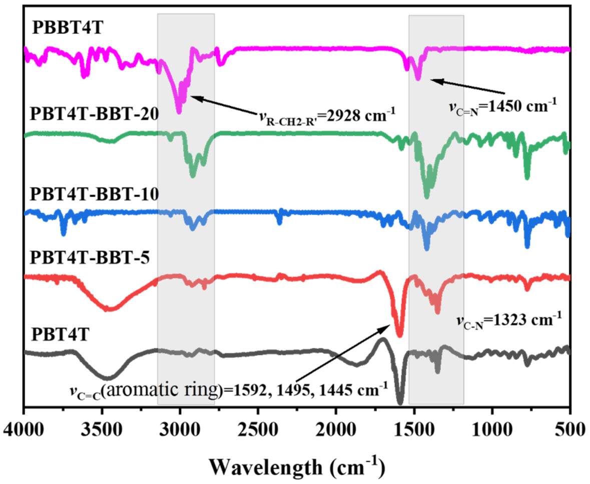

Figure S1. Fourier infrared spectra of PBT4T, PBT4T-BBT-5, PBT4T-BBT-10, PBT4T-BBT-20 and PBBT4T.

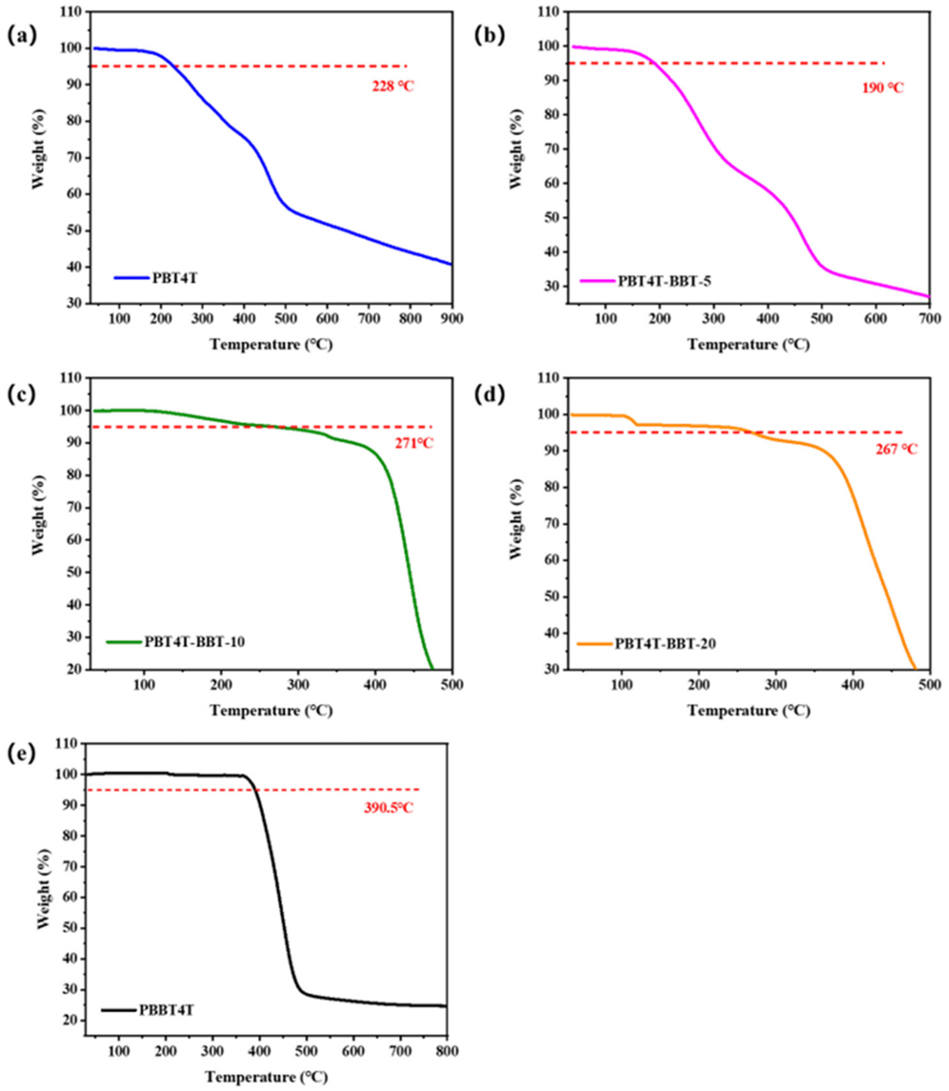

Figure S2. The TGA curves of PBT4T, PBT4T-BBT-5, PBT4T-BBT-10, PBT4T-BBT-20 and PBBT4T.

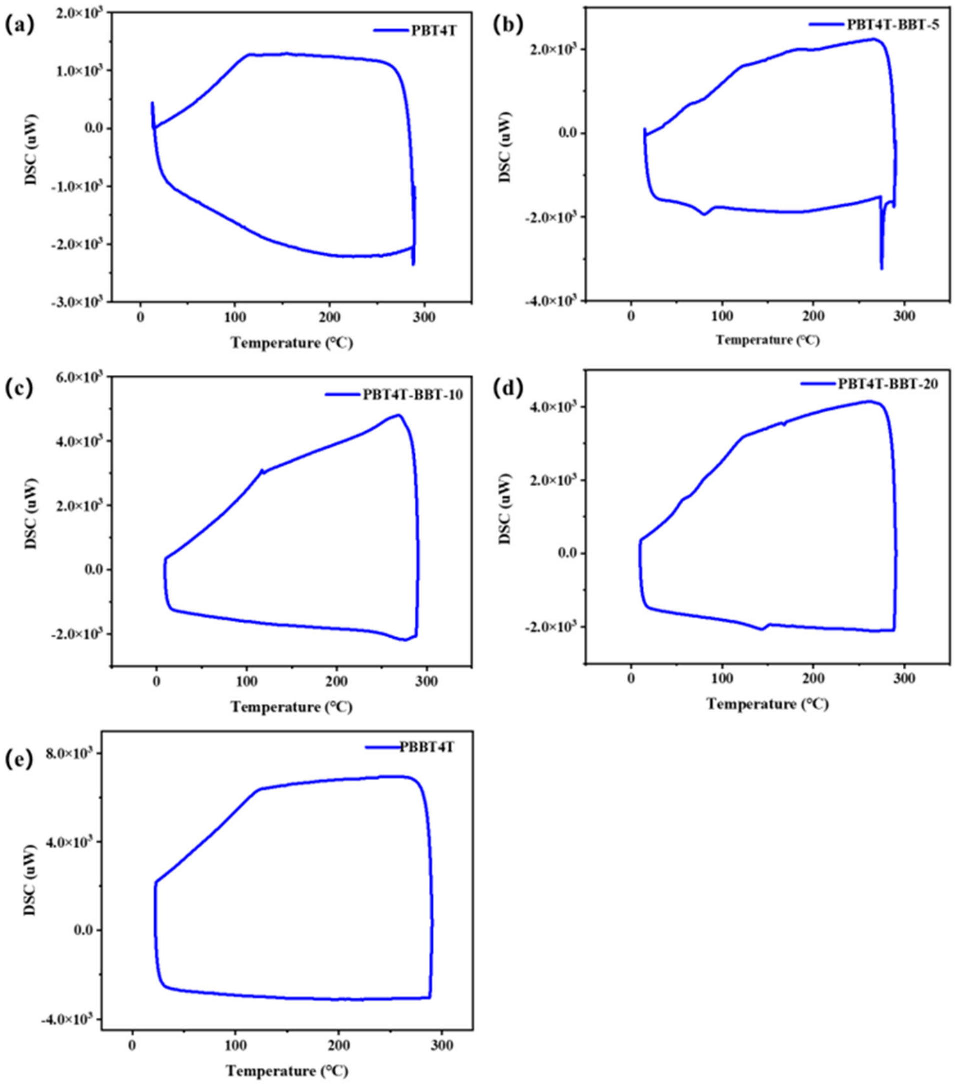

Figure S3. DSC curves of PBT4T, PBT4T-BBT-5, PBT4T-BBT-10, PBT4T-BBT-20 and PBBT4T.

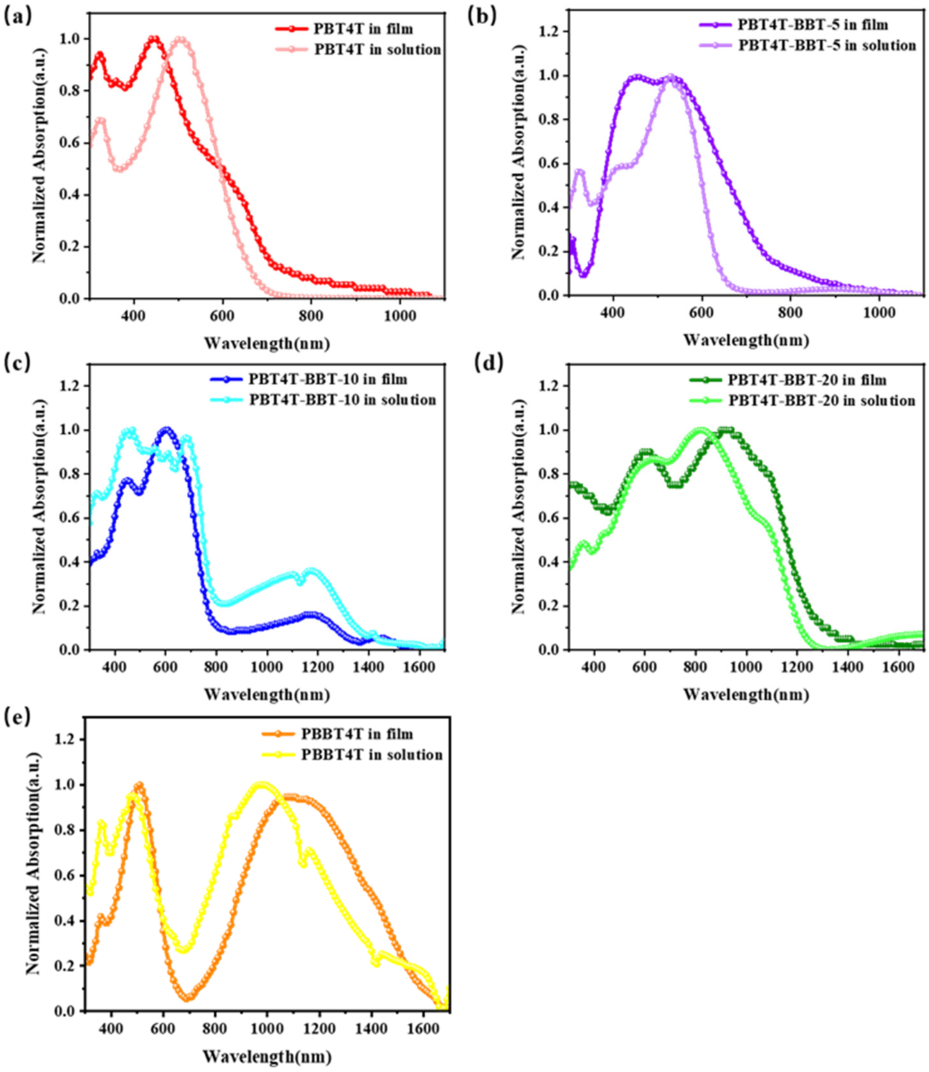

Figure S4. The UV-vis absorption curves of PBT4T, PBT4T-BBT-5, PBT4T-BBT-10, PBT4T-BBT-20 and PBBT4T in film and solution.

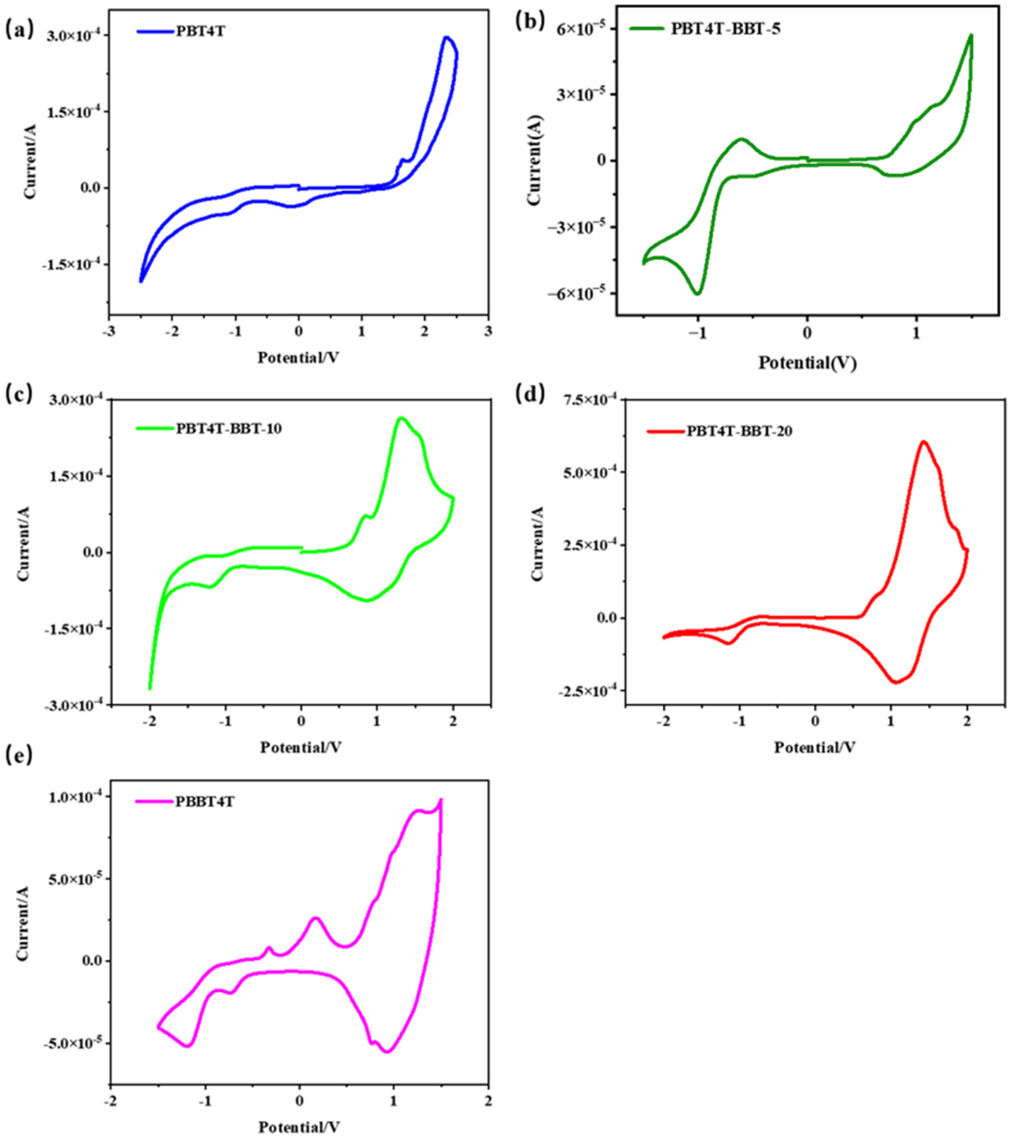

Figure S5. The CV curves of PBT4T, PBT4T-BBT-5, PBT4T-BBT-10, PBT4T-BBT-20 and PBBT4T.

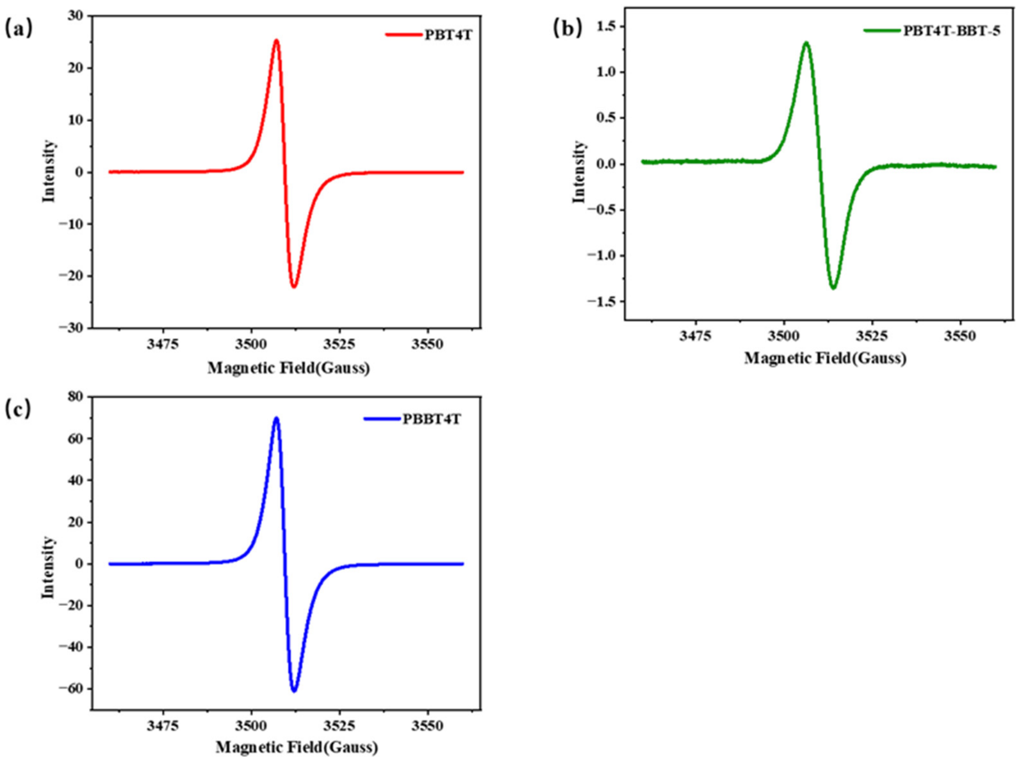

Figure S6. ESR spectrum of (a) PBT4T, (b) PBT4T-BBT-5 and (c) PBBT4T.

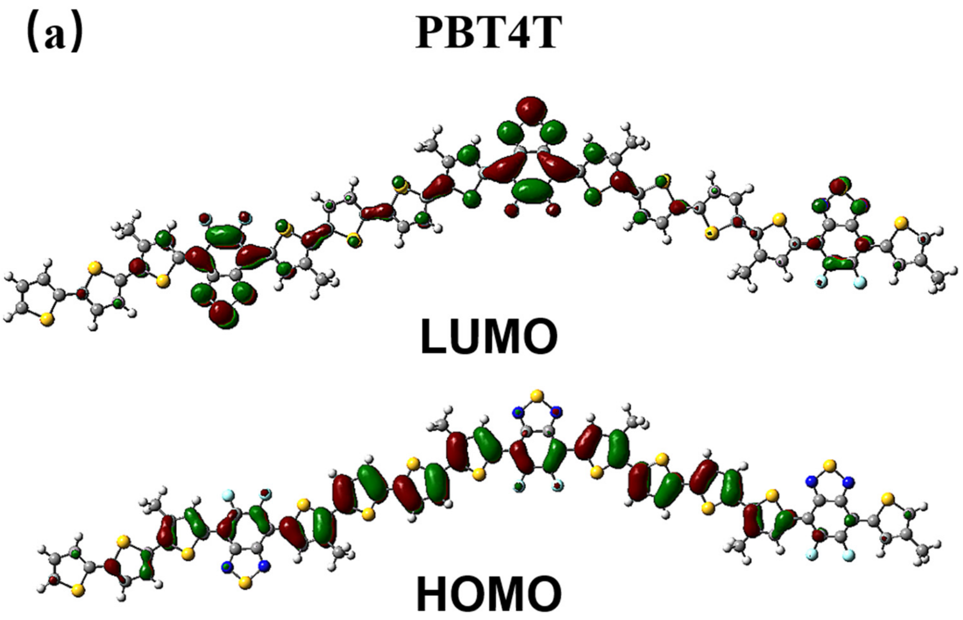

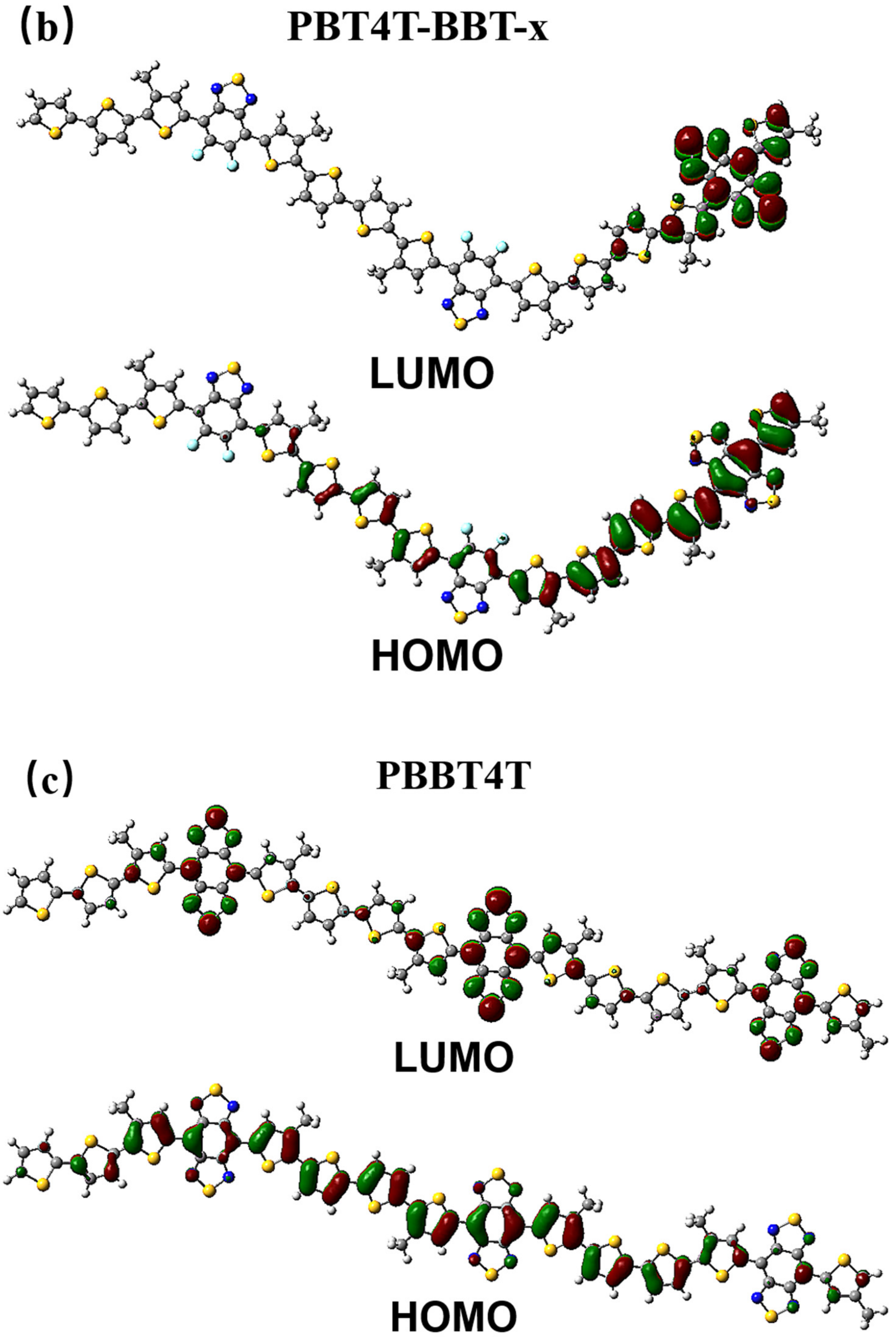

Figure S7. HOMO and LUMO energy level diagrams of (a) PBT4T, (b) PBT4T-BBT-x and (c) PBBT4T.

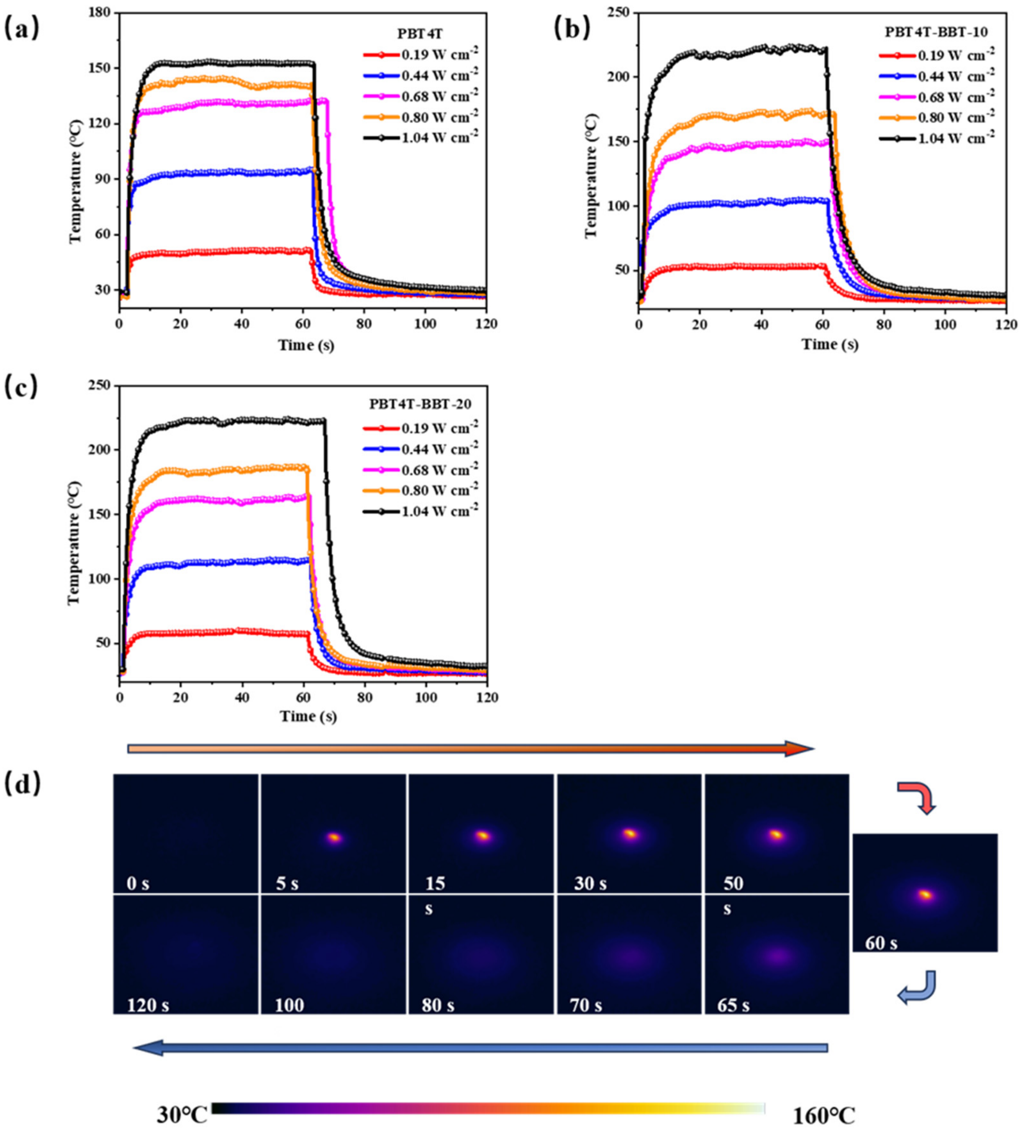

Figure S8. (a) PBT4T, (b) PBT4T-BBT-10, (c) PBT4T-BBT-20 powder under 808nm laser irradiation at different power irradiation, (d) Infrared thermal imaging under 808 nm laser at 0.68 W cm^{-2} of PBT4T-BBT-5 powder.

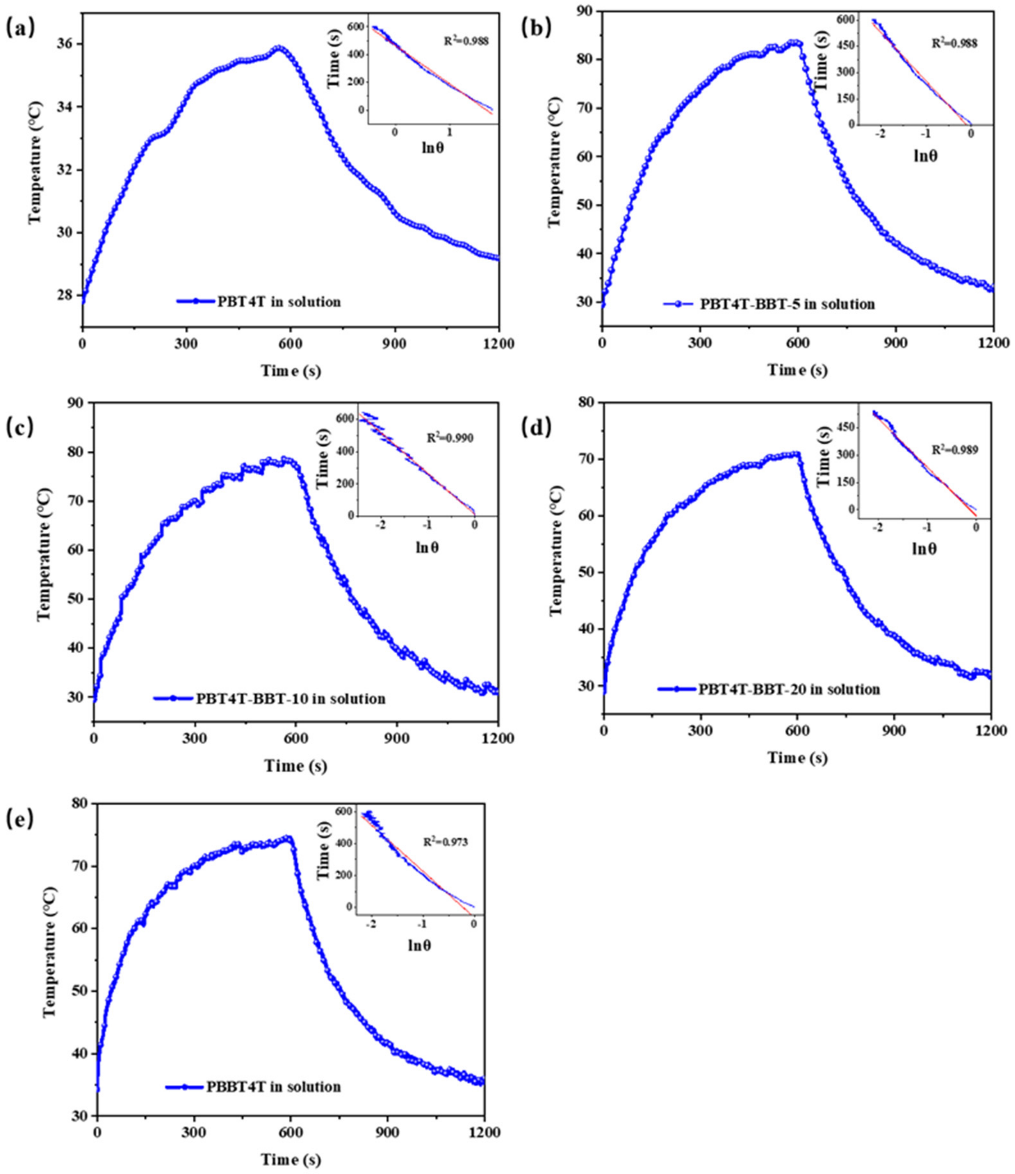

Figure S9. Photothermal properties of (a) PBT4T, (b) PBT4T-BBT-5, (c) PBT4T-BBT-10, (d) PBT4T-BBT-20 and (e) PBBT4T under 808nm laser irradiation (1 W cm^{-2}). The illustration shows the time-*ln* θ linear curve of the corresponding cooling process.

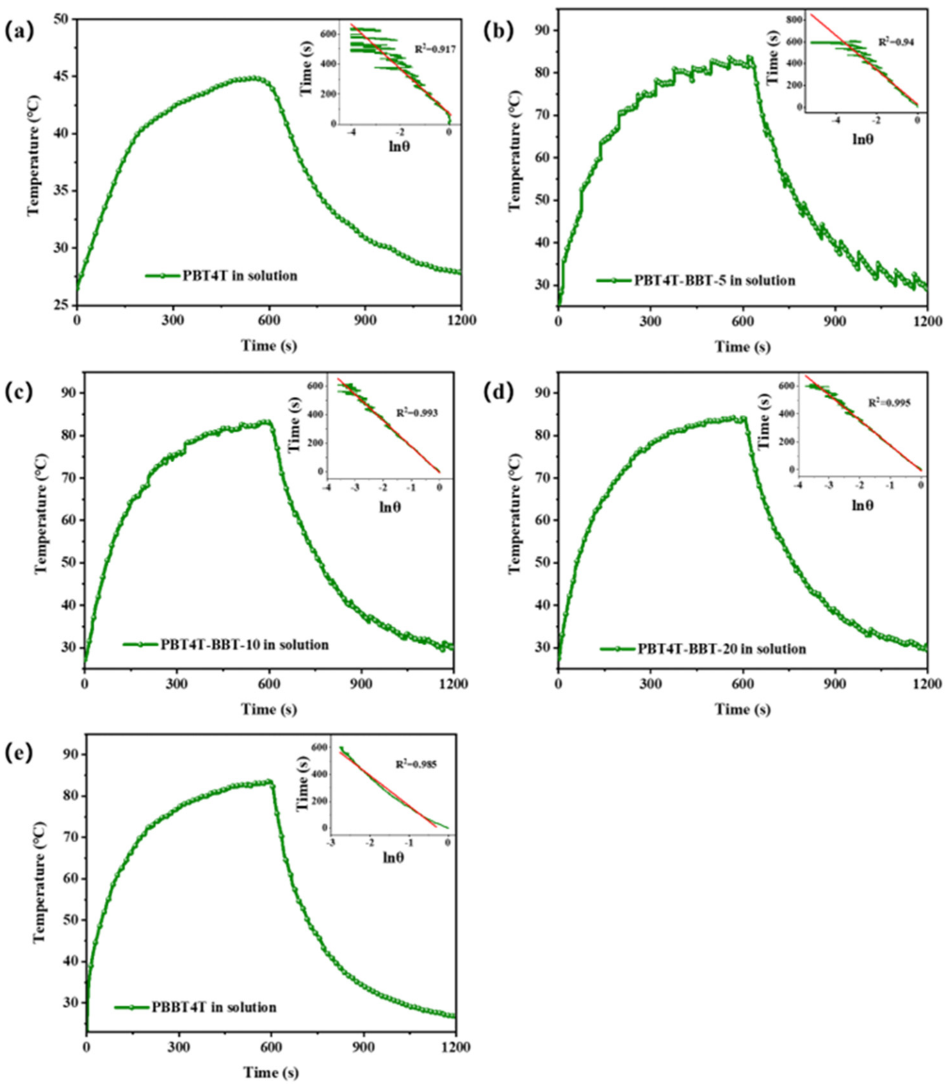

Figure S10. Photothermal properties of (a) PBT4T, (b) PBT4T-BBT-5, (c) PBT4T-BBT-10 (d) PBT4T-BBT-20 and (e) PBBT4T under 980 nm laser irradiation (1 W cm^{-2}). The illustration shows the time-*ln θ* linear curve of the corresponding cooling process.

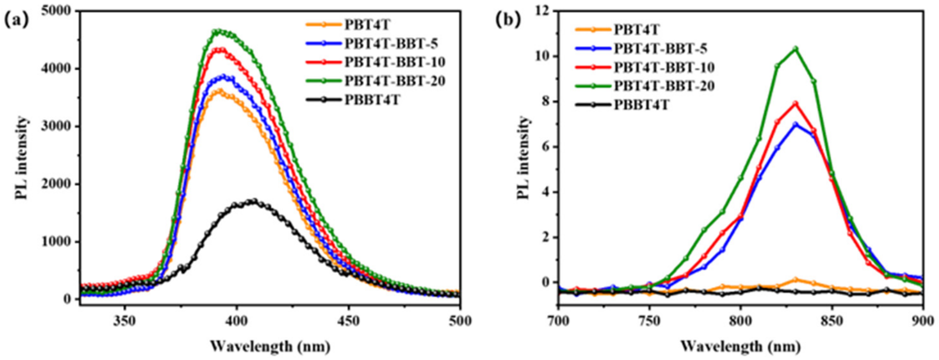

Figure S11. Fluorescence spectra at (a) 638 nm and (b) 980 nm excitation wavelength of PBT4T, PBT4T-BBT-5, PBT4T-BBT-10, PBT4T-BBT-20 and PBBT4T.

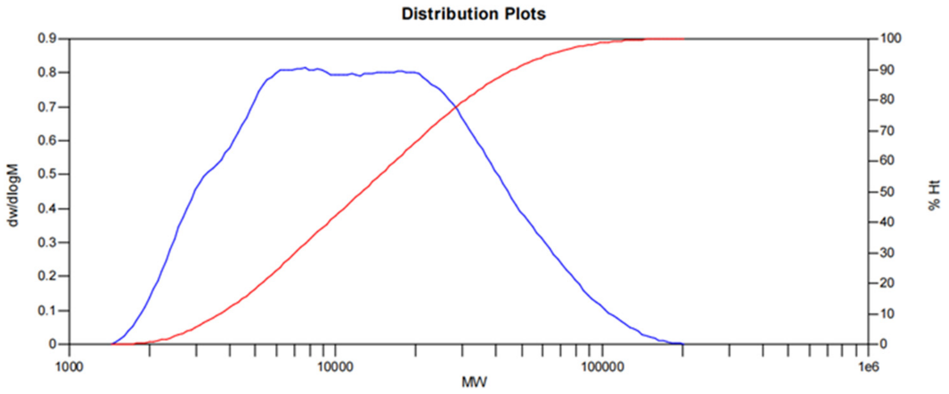

Figure S12. GPC trace of PBBT4T.

Table S1. GPC trace analysis of PBBT4T in Figure S12.

MW Averages

Peak No	Mp	Mn	Mw	Mz	Mz+1	Mv	PD
1	7686	8381	19837	42462	68725	17394	2.3669

Processed Peaks

Peak No	Start RT (mins)	Max RT (mins)	End RT (mins)	Pk Height (mV)	%Height	Area (mV.secs)	%Area
1	7.08	8.68	9.35	8.83934	0	691.232	100

Table S2. GPC trace analysis of PBT4T.

MW Averages

Peak No	Mp	Mn	Mw	Mz	Mz+1	PD
1	3381	2107	3961	6383	9565	1.879924

Processed Peaks

Peak No	Start RT (mins)	Max RT (mins)	End RT (mins)	Area (mV.secs)	%Area
1	7.818	8.938	10.302	610194.0168	100

Table S3. GPC trace analysis of PBT4T-BBT-10.

MW Averages

Peak No	Mp	Mn	Mw	Mz	Mz+1	PD
1	4715	1511	4284	9050	14548	2.835208

Processed Peaks

Peak No	Start RT (mins)	Max RT (mins)	End RT (mins)	Area (mV.secs)	%Area
1	7.504	8.615	10.103	109041.0014	100

References

1. Zhang, R.; Jin, N.; Jia, T.; Wang, L.; Liu, J.; Nan, M.; Qi, S., Liu, S.; Pan, Y. A narrow-bandgap photothermal material based on a donor–acceptor structure for the solar–thermal conversion application. J. Mater. Chem. A. 2023, 11, 15380.
